# Adaptation of Risk Score for Hepatocellular Carcinoma Without Alcohol Measures

**DOI:** 10.1001/jamanetworkopen.2025.22305

**Published:** 2025-07-22

**Authors:** Janet P. Tate, Tamar H. Taddei, Vincent Lo Re, Amy C. Justice

**Affiliations:** 1 Department of Internal Medicine, Yale School of Medicine, New Haven, Connecticut; 2 Department of Medicine, Veterans Affairs Connecticut Healthcare, West Haven, Connecticut; 3 Division of Infectious Diseases, Department of Medicine, and Center for Clinical Epidemiology and Biostatistics, Department of Biostatistics, Epidemiology, and Informatics, Perelman School of Medicine, University of Pennsylvania, Philadelphia; 4 Department of Health Policy and Management, Yale School of Public Health, New Haven, Connecticut

## Abstract

This diagnostic study validates hepatocellular carcinoma risk scores without alcohol measures in veterans with and without alcohol use disorder.

## Introduction

Liver disease is often missed in the primary care setting. The Fibrosis-4 Index (FIB-4) is a first-line assessment for receipt of frequent or intensive monitoring.^1,2,3,4,5^ Ilagan-Yin et al^6^ showed that a multivariable risk score based on age, sex, race and ethnicity, liver aminotransferases, platelets, diabetes, smoking, alcohol use patterns, and body mass index (BMI) outperformed FIB-4 in identifying patients at risk of hepatocellular carcinoma (HCC). The score differentiated risk within FIB-4 levels, particularly in individuals with obesity, diabetes, unhealthy alcohol use, smoking, and Hispanic ethnicity. The HCC risk score was developed and internally validated in more than 6 million US veterans, with a critical next step to validate it outside this population. However, some cohorts that could otherwise be used for validation do not collect Alcohol Use Disorders Identification Test–Consumption (AUDIT-C) data. History of alcohol use disorder (AUD) also may not be captured uniformly. Thus, we developed risk scores that first omit AUDIT-C and then any alcohol measure.

## Methods

This diagnostic study used previously described data and statistical analyses.^6^ The Yale University and VA Connecticut Healthcare System institutional review boards approved the study and waived informed consent as data were collected as part of routine care. We followed the TRIPOD reporting guideline.

We collected covariate data at the time of visit between October 1, 2007, and March 30, 2020, and followed up patients until December 31, 2021, for incident HCC. Veterans aged 30 to 95 years were included, but those with hepatitis B or C, hepatic decompensation, or prevalent HCC were excluded. Race and ethnicity, known to be associated with HCC risk, were self-reported (Black, Hispanic, White, other [American Indian or Alaska Native, Asian, Native Hawaiian or Pacific Islander, multiracial]). Alcohol use was classified as an AUD diagnosis before the index date or, in the absence of AUD, as assessed using AUDIT-C and categorized as abstinent or low, moderate, or high risk.

We used Cox regression to develop risk scores in a development sample and evaluated performance in a validation sample and several subgroups. We compared 3 models: (1) using the full alcohol classification, (2) omitting AUDIT-C, and (3) further omitting AUD. A 2-sided *P* < .05 was considered significant. Data were analyzed using SAS, version 9.4 (SAS Institute Inc).

## Results

The sample included 6 509 288 veterans (median [IQR] age, 65 [53-74] years; 7% female and 93% male), with 5 119 775 in the development and 1 389 513 in the validation cohort. During follow-up, 10 896 HCC events occurred in the development and 4246 in the validation cohort.^6^ Alcohol use disorder was present in 13.8%, abstinence in 43.3%, and high-risk alcohol consumption in 1.0%; AUD was more common in current smokers (26.3%) and veterans with BMI less than 20 (25.9%). The AUD hazard ratio (HR) was 1.62 (95% CI, 1.53-1.72) in model 1, decreasing to 1.51 (95% CI, 1.43-1.59) in model 2 (Table). Model 1 associations with current smoking (HR, 1.71; 95% CI, 1.62-1.81) were maintained in model 2 but increased in model 3 (HR, 1.82; 95% CI, 1.73-1.92). Subtle differences in coefficients used to calculate risk scores were seen with alanine aminotransferase and BMI. The distribution of scores, discrimination (C statistic, 0.83; 95% CI, 0.81-0.84), and predicted HCC risk vs score were similar across models. Observed HCC risk matched overall and subgroup predictions by FIB-4 level. However, in model 3, risk was slightly underestimated in patients with previous AUD (Figure).

**Table.** Cox Proportional Hazards Regression Model Fit to Development Sample of 5 119 775 Veterans With 10 896 Hepatocellular Carcinoma Events During a Maximum 10-Year Follow-Up

Covariate^a^	Model 1, AUD and AUDIT-C				Model 2, AUD only				Model 3, no alcohol measure			
	PE	χ^2^	*P* value	HR (95% CI)	PE	χ^2^	*P* value	HR (95% CI)	PE	χ^2^	*P* value	HR (95% CI)
Age, years												
*X* = (age − 50) / 5	0.782	627	<.001	2.19 (2.06-2.32)	0.786	634	<.001	2.19 (2.06-2.33)	0.790	643	<.001	2.20 (2.07-2.34)
*X*^2^	−0.083	76	<.001	0.92 (0.90-0.94)	−0.083	78	<.001	0.92 (0.90-0.94)	−0.088	88	<.001	0.92 (0.90-0.93)
*X*^3^	0.002	4	.03	1.00 (1.00-1.00)	0.002	5	.026	1.00 (1.00-1.00)	0.002	7	.007	1.00 (1.00-1.00)
Sex												
Female	−0.616	50	<.001	0.54 (0.46-0.64)	−0.608	49	<.001	0.54 (0.46-0.65)	−0.647	55	<.001	0.52 (0.44-0.62)
Male	NA	NA	NA	1 [Reference]	NA	NA	NA	1 [Reference]	NA	NA	NA	1 [Reference]
Race and ethncity												
Black, non-Hispanic	−0.472	144	<.001	0.62 (0.58-0.67)	−0.464	140	<.001	0.63 (0.58-0.68)	−0.440	126	<.001	0.64 (0.60-0.70)
Hispanic	0.566	242	<.001	1.76 (1.64-1.89)	0.571	246	<.001	1.77 (1.65-1.90)	0.593	266	<.001	1.81 (1.69-1.94)
White, non-Hispanic	NA	NA	NA	1 [Reference]	NA	NA	NA	1 [Reference]	NA	NA	NA	1 [Reference]
Other^b^	0.049	1	.28	1.05 (0.96-1.15)	0.058	2	.20	1.06 (0.97-1.16)	0.071	2	.12	1.07 (0.98-1.17)
Unknown	0.204	33	<.001	1.23 (1.14-1.32)	0.200	32	<.001	1.22 (1.14-1.31)	0.191	29	<.001	1.21 (1.13-1.30)
FIB-4 components												
ALT, IU/L												
*X* = (ALT − 30) / 10	−0.065	32	<.001	0.94 (0.92-0.96)	−0.068	35	<.001	0.93 (0.91-0.96)	−0.073	40	<.001	0.93 (0.91-0.95)
*X*^2^	0.002	0	.65	1.00 (0.99-1.01)	0.003	0	.51	1.00 (0.99-1.01)	0.003	0	.54	1.00 (0.99-1.01)
*X*^3^	−0.001	2	.19	1.00 (1.00-1.00)	−0.001	2	.15	1.00 (1.00-1.00)	−0.001	2	.13	1.00 (1.00-1.00)
AST, IU/L												
*X* = (AST − 30) / 10	0.720	2834	<.001	2.05 (2.00-2.11)	0.721	2844	<.001	2.06 (2.00-2.11)	0.735	2967	<.001	2.08 (2.03-2.14)
*X*^2^	−0.067	241	<.001	0.94 (0.93-0.94)	−0.067	243	<.001	0.94 (0.93-0.94)	−0.066	232	<.001	0.94 (0.93-0.94)
*X*^3^	0.002	30	<.001	1.00 (1.00-1.00)	0.002	31	<.001	1.00 (1.00-1.00)	0.002	26	<.001	1.00 (1.00-1.00)
PLT, per μL												
*X* = (PLT − 200) / 50	−0.383	907	<.001	0.68 (0.67-0.70)	−0.383	906	<.001	0.68 (0.67-0.70)	−0.386	923	<.001	0.68 (0.66-0.70)
*X*^2^	0.168	2098	<.001	1.18 (1.17-1.19)	0.169	2120	<.001	1.18 (1.18-1.19)	0.171	2190	<.001	1.19 (1.18-1.20)
*X*^3^	−0.011	48	<.001	0.99 (0.99-0.99)	−0.011	49	<.001	0.99 (0.99-0.99)	−0.012	50	<.001	0.99 (0.99-0.99)
Diabetes												
No	NA	NA	NA	1 [Reference]	NA	NA	NA	1 [Reference]	NA	NA	NA	1 [Reference]
Yes	0.883	1871	<.001	2.42 (2.32-2.52)	0.900	1969	<.001	2.46 (2.36-2.56)	0.884	1908	<.001	2.42 (2.33-2.52)
Smoking												
Never	NA	NA	NA	1 [Reference]	NA	NA	NA	1 [Reference]	NA	NA	NA	1 [Reference]
Current	0.537	395	<.001	1.71 (1.62-1.81)	0.530	387	<.001	1.70 (1.61-1.79)	0.598	509	<.001	1.82 (1.73-1.92)
Former	0.171	50	<.001	1.19 (1.13-1.24)	0.161	45	<.001	1.18 (1.12-1.23)	0.173	52	<.001	1.19 (1.14-1.25)
Alcohol^c^												
AUD	0.485	251	<.001	1.62 (1.53-1.72)	0.409	245	<.001	1.51 (1.43-1.59)	NA	NA	NA	NA
Abstinent	0.147	37	<.001	1.16 (1.11-1.21)	NA	NA	NA	NA	NA	NA	NA	NA
Low risk	NA	NA	NA	1 [Reference]	NA	NA	NA	NA	NA	NA	NA	NA
Moderate risk	−0.063	2	.12	0.94 (0.87-1.02)	NA	NA	NA	NA	NA	NA	NA	NA
High risk	0.202	4	.03	1.22 (1.02-1.48)	NA	NA	NA	NA	NA	NA	NA	NA
BMI												
*X* = (BMI − 25) / 5	0.165	66	<.001	1.18 (1.13-1.23)	0.162	64	<.001	1.18 (1.13-1.22)	0.141	48	<.001	1.15 (1.11-1.20)
*X*^2^	0.023	2	.13	1.02 (0.99-1.05)	0.026	3	.08	1.03 (1.00-1.06)	0.033	5	.03	1.03 (1.00-1.06)
*X*^3^	−0.008	8	.005	0.99 (0.99-1.00)	−0.009	9	.003	0.99 (0.99-1.00)	−0.010	11	.001	0.99 (0.98-1.00)

Abbreviations: ALT, alanine aminotransferase; AST, aspartate aminotransferase; AUD, alcohol use disorder; BMI, body mass index (calculated as weight in kilograms divided by height in meters squared); FIB-4, Fibrosis-4 Index; HR, hazard ratio; NA, not applicable; PE, parameter estimate; PLT, platelets.

^a^ *X* is the transformation of the variable into its centered value on a meaningful scale. *X*^2^ is the square of this value, and *X*^3^ is the cube. For example, age is centered at 50 years and then divided by 5 so that the HR represents the association of a 5-year increment of age older or younger than 50 years.

^b^ Other race and ethnicity included American Indian or Alaska Native, Asian, Native Hawaiian or Other Pacific Islander, and multiracial.

^c^ Categories in the absence of AUD, by Alcohol Use Disorders Identification Test–Consumption, are abstinent (0 points), low risk (1-2 points for female or 1-3 points for male), moderate risk (3-7 points for female or 4-7 points for male), and high risk (≥8 points for female and male).

**Figure.**
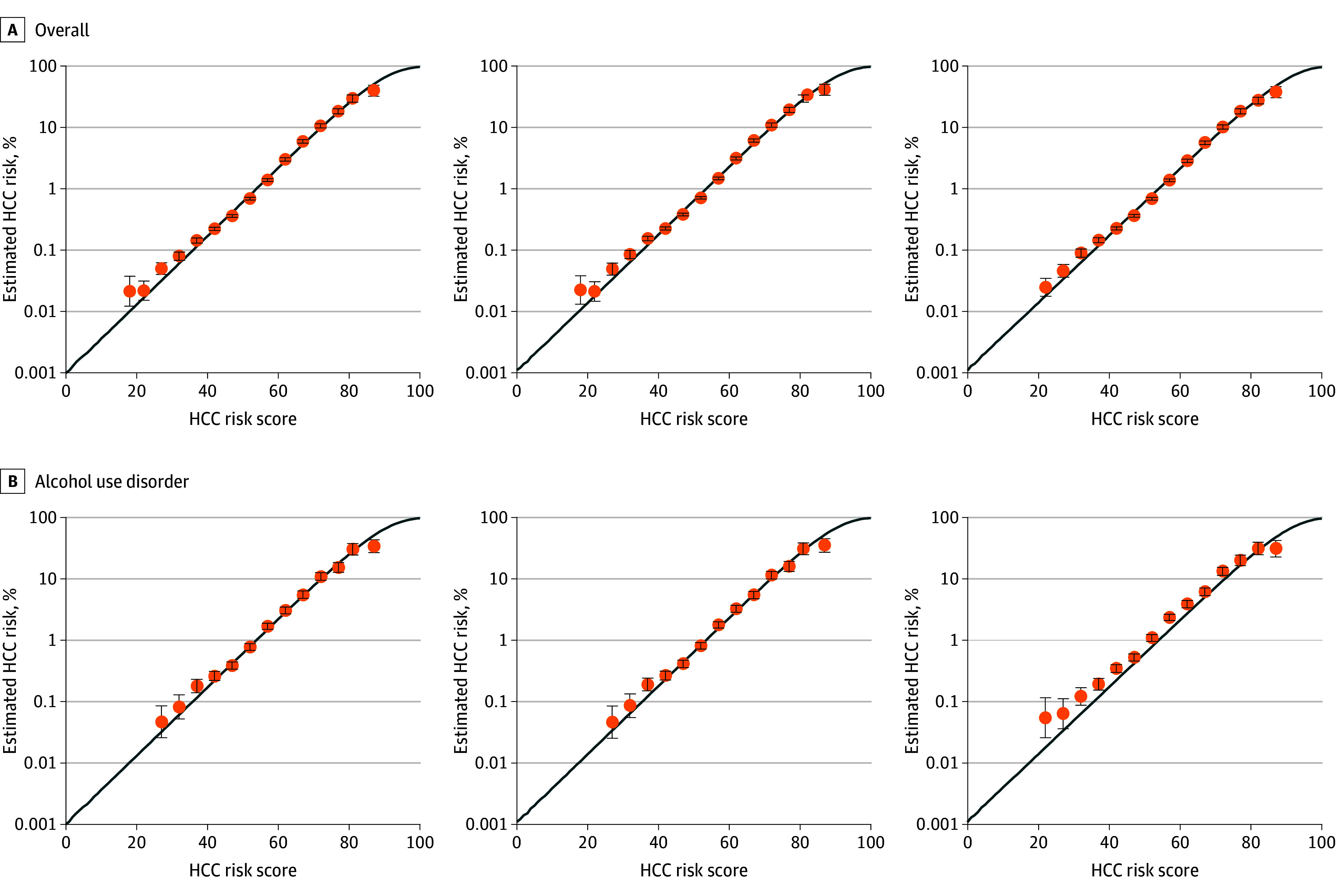
10-Year Risk of Hepatocellular Carcinoma (HCC) Model 1 uses the full alcohol classification, including Alcohol Use Disorders Identification Test–Consumption (AUDIT-C) and alcohol use disorder; model 2 omits AUDIT-C; and model 3 omits both AUDIT-C and alcohol use disorder. Lines were estimated using risk scores obtained from the sample. Subgroup data points from Kaplan-Meier estimates are shown for a minimum of 10 patients with HCC events and 5 who were at risk for HCC at the end of follow-up. Error bars represent 95% CIs.

## Discussion

This diagnostic study shows that omitting alcohol measures did not substantively change performance of the HCC risk score. Covariates known to be associated with AUD were more important when alcohol measures were omitted. Measurement of alcohol use is imperfect since AUDIT-C relies on self-report and AUD may be differentially documented. Although other health systems may capture current AUD, prior history may be incomplete. This work is limited by a population of predominantly White, middle-aged, male veterans. These alternative models may allow validation and implementation in a wider range of cohorts.

## Notes

Supplement.
Data Sharing Statement
