# Supplementary material for: Adaptation of Risk Score for Hepatocellular Carcinoma Without Alcohol Measures
Source: JAMA Netw Open. 2025 Jul 22;8(7):e2522305. doi: 10.1001/jamanetworkopen.2025.22305 (PMC12284736; doi:10.1001/jamanetworkopen.2025.22305)
Supplement: Supplement. — Data Sharing Statement [file jamanetwopen-e2522305-s001.pdf]

Data Sharing Statement

Tate. Adaptation of Risk Score for Hepatocellular Carcinoma Without Alcohol Measures. *JAMA Netw Open*. Published July 22, 2025. doi:10.1001/jamanetworkopen.2025.22305

Data
Data available: No

Additional Information
Explanation for why data not available: The data that support the findings of this study are not permitted to leave the VA firewall without a Data Use Agreement due to VA regulations. However, VA data are made freely available to researchers with an approved VA study protocol. For more information, please contact the VA Information Resource Center (VIReC) at VIRec@ va.gov or the corresponding author.
